# Pet Owners’ Perceptions of Key Factors Affecting Animal Welfare During Veterinary Visits

**DOI:** 10.3390/ani15060894

**Published:** 2025-03-20

**Authors:** Andrei-Sebastian Csiplo, Silvana Popescu

**Affiliations:** 1 Department of Animal Hygiene and Welfare, Faculty of Veterinary Medicine, University of Agricultural Sciences and Veterinary Medicine Cluj-Napoca, 400372 Cluj-Napoca, Romania; silvana.popescu@usamvcluj.ro; 2 Centre for Animal Welfare, University of Veterinary Medicine Budapest, H-1078 Budapest, Hungary

**Keywords:** companion animals, behavior, veterinary consultation, stress, owners’ perception, welfare status

## Abstract

Over the past decade, the number of companion dogs and cats has grown significantly, leading to an increase in veterinary visits. These visits can be stressful for animals, potentially affecting their long-term physical and emotional well-being. The aim of this study was to investigate how dog and cat owners perceive the factors influencing their pets’ behavior and welfare during veterinary visits. After completing a veterinary visit, dog and cat owners filled a survey assessing their pets’ behavioral responses at different stages of the consultation. A total of 94 dog and cat owners participated in this study. The findings revealed that perceived stress levels were the highest right after entering the practice but declined significantly after about 10 min in the waiting room. The owners’ observations, based on their familiarity with their pets’ typical behavior, were considered a useful tool for assessing the stress levels in their companion animals during the veterinary visits. Although they had the possibility to choose terms from a list of behaviors, no ethogram was provided previously or during the data collection. In conclusion, while stress remains a key concern for animal welfare, the most critical period identified was immediately before and after entering the veterinary practice. Further research is needed to better understand this crucial phase and its implications for the health and welfare of dogs and cats.

## Simple Summary

Nowadays, alongside the growing number of dog and cat owners, the awareness for the welfare of these animals is increasing society-wide. Because of the time spent together in spatial proximity and because they often have shared activities, owners could be the best observers of the factors impacting the well-being of their animals. Although visits for veterinary treatments are regarded as stressful events for pets (and because of this, oftentimes, for their owners too), owners’ opinions of these situations have not been systematically researched. This study aimed to explore dog and cat owners’ perceptions of key factors influencing their emotional state and, thus, the welfare of their animals. The insight given by this paper could contribute to lowering pets’ stress during veterinary interventions, both by animal owners and veterinary personnel.

## 1. Introduction

During the last decade, the number of pet-owning households has increased by approximately 20 million, and as of 2023, an estimated 166 million European households own at least one pet [1]. According to the latest data, the European Union has a population of more than 72 million dogs and 83 million cats, and 74% of Europeans hope for better protection of companion animal welfare in their countries [2]. Matching this trend, all related services and product industries have also grown, including the number and standards of veterinary providers. Owners well recognize the importance of proper health care in their companion animals. Yet, multiple studies show that, when visiting a veterinarian, dogs and cats often display signs of fear and stress [3], passively influencing owners’ behavior as well [4]. According to the same authors, 28% of cat and 22% of dog owners would visit a veterinarian more regularly for a routine check-up if their pet did not suffer the effects of stress during visits. The intensity of fear and distress is shaped by the animal’s personality and previous experiences, but the veterinary setting itself can introduce multiple stress triggers. Among these, previous studies mention confinement means of the general practice, clinic, or hospital [5], transport to the medical facility [6], hostility or novelty of the environment [7], and the use of harsh handling techniques during consultation [8]. Unfortunately, fear and anxiety often lead to physiological stress responses in animals exposed to these conditions [9]. These stress responses can trigger various hormonal and immune modulatory processes, potentially shortening the lifespan of animals [9]. Therefore, veterinarians must consider their patients’ emotional state and well-being when performing painful procedures (e.g., vaccination) to minimize stress and safeguard their welfare [10].

Most farm species, such as dairy cattle, pigs, and poultry, are already evaluated through established animal welfare evaluation systems (e.g., Welfare Quality^®^). Despite the strong link between veterinary care and animal welfare, no comparable system currently exists for companion animals in veterinary facilities [11]. To lay the groundwork for such a system, researchers and professionals have surveyed key characteristics of veterinary care that may influence the welfare of companion animals [11]. These include recognizing animal signals, applying appropriate handling and restraint methods, and providing owners with guidance on their pet’s behavior at home [11]. However, to support owners’ understanding of companion animal behavior and welfare, several international organizations (e.g., The International Society of Feline Medicine, The Online Dog Trainer) have developed guidelines and educational materials [12].

This study aims to investigate dog and cat owners’ perceptions of the factors influencing their pets’ behavior and welfare during veterinary visits. Our findings contribute to the body of research into the behavior and welfare of companion animals from their owners’ perspective, supporting the need for a standardized welfare assessment protocol for dogs and cats during veterinary consultations, given the increasing interest in pet ownership and veterinary medical services over the last years.

## 2. Materials and Methods

### 2.1. Owner Questionnaire

The first step of this study was to develop a comprehensive questionnaire (see Supplementary Materials) for the companion animal owners to collect a broad range of data at the completion of a veterinary visit with their pet(s). Out of the 47 survey items, some were general (asking for participation, image capturing, and personal data usage agreement, date, reason for visiting the practice, basic information about the pets). The next part explored the home environment of the pets (outdoor access, company of the same or other species, owner–animal interactions). One of the most important sections focused on the behaviors observed by the owner, at three key moments of the veterinary visit: at their arrival at the practice, right after entering, and after about 10 min spent in the waiting room. The owners had the possibility to choose from 19 behaviors listed as examples. Then, they had to rate on a Likert scale (from 1 to 5, where 1 was inexistent and 5 was extreme) the stress level of their animal in the same three moments, then during subsequent stages of the consultation (weighing, entering the consultation room, veterinary approach, and animal manipulation by the personnel, during the consultation). Another list of 19 items was provided to the owners to first identify the daily behaviors of their pets in their home environment, then to establish the five most frequent patterns observed at the veterinary clinic. For the latter, they also had to assign a level of intensity (from 1, non-existent, to 10, very obvious). Other questions explored the owners’ opinion on factors impacting small animal welfare during veterinary consultations and possibilities to limit that.

Upon their entrance into the waiting room, the owners were presented with the scope and content of this study, briefly, asking for their willingness to participate. If they agreed, they were asked if they paid attention to the behavior of their animal(s) during their commute to the practice and at the entrance (right before and right after entering). If they replied with “Yes”, they were asked to pay attention to their pet’s behavior after about ten minutes, at their weighing, before entering the consultation room, and until the end of their visit at the practice, at any time they were near their animal(s). Then, before they were about to leave the practice, they were asked to fill the questionnaire.

The questionnaire was available both online (accessible by a QR code) and on paper, at the choice of the respondents, once the veterinary practice visit was complete. Through these methods, a total of 94 questionnaires were filled for further processing. All data were anonymized in line with Regulation (EU) 2016/679 [13] on the protection of natural persons.

Besides the owners’ agreement, the study inclusion criteria regarded the animals as well. To be included in this study, they had to be clinically healthy, according to the assessment of the veterinary personnel of the facility.

### 2.2. Data Processing and Statistical Analysis

All data provided by the questionnaires (both in the online and paper-based versions) were transferred to an Excel (Microsoft Office 2021 Pro-Plus) file. The frequency of answers was established for each question by counting them (absolute frequencies, i.e., counting the answers for the multiple-choice Likert-scale-type questions) and/or calculating percentages (by dividing the frequency of a given answer with the total number of possible answers, for example, for the questions about animal behaviors). This kind of post-processing allowed the transformation of some data from qualitative to quantitative. Some open-ended questions recorded the opinion of the owners, and quotes were collected, but these were not used in this study. 

All data recorded by the surveys (on online Google Forms documents) were stored in a Google Drive file. Data analysis was performed by using SPSS (version 17, SPSS Inc., Chicago, IL, USA) software. The owner-provided results were expressed as percentages and compared to the most prevalent behaviors reported at different stages of consultation. The normality distribution of these data was tested by the Kolmogorov–Smirnov test, whilst Friedman and subsequent Wilcoxon tests were used for comparisons. Differences were considered statistically significant at a *p*-value < 0.05.

## 3. Results

### 3.1. Common Reasons for a Veterinary Visit

**Figure 1:**
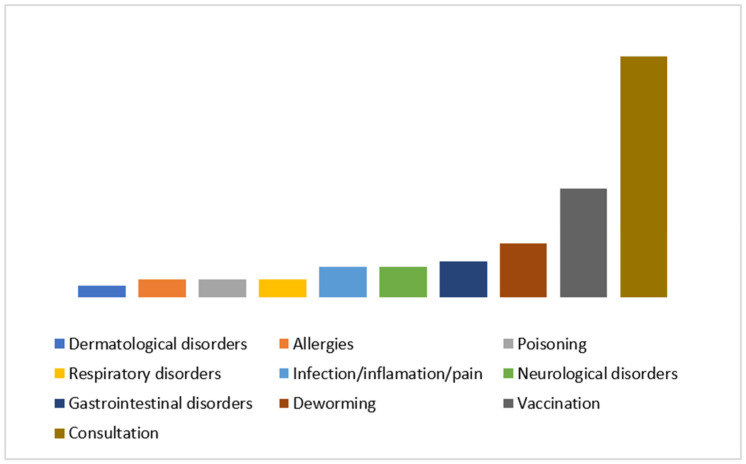
Distribution of reasons for visiting a veterinarian within the group of the 94 study subjects.

The results of this study entirely reflect the owners’ perceptions about their animals’ behavior. Out of a total of 94 respondents, more than half (58.51%) were dog owners, and 41.49% owned cats. A considerable proportion of owners (42.55%) indicated that they had an appointment for a regular health check-up (or consultation), 19.15% went for their pet’s routine vaccination, and 9.57% went for a routine deworming treatment. The most frequently declared pet health issues included gastrointestinal (6.38%), neurological (5.32%), respiratory (3.19%), and dermatological (2.13%) problems. A small proportion of animals (5.32%) showed obvious signs of infection, inflammation, or pain, and approximately 3.19% had poisoning symptoms (Figure 1). Only 3.19% of the animals were suspected of having allergies or visited the veterinarian for allergy-related follow-up consultations. While other scientific studies do not describe the reasons for visiting the veterinarian, this study highlighted some of the most frequent causes. This categorization gave an insight into the animals visiting a medical facility and on the typology of clients.

### 3.2. Age and Gender Distribution of the Studied Animals

According to their gender and age, the studied dogs and cats were grouped in categories and subcategories (Table 1).

### 3.3. Housing Characteristics and Social Interaction

Housing- and social interaction-related data were collected to get a better insight into all factors that could potentially affect the behavior of animals in a known environment (e.g., home). The prevalence of each situation is shown in Table 2.

### 3.4. Identification of Stress

**Figure 2:**
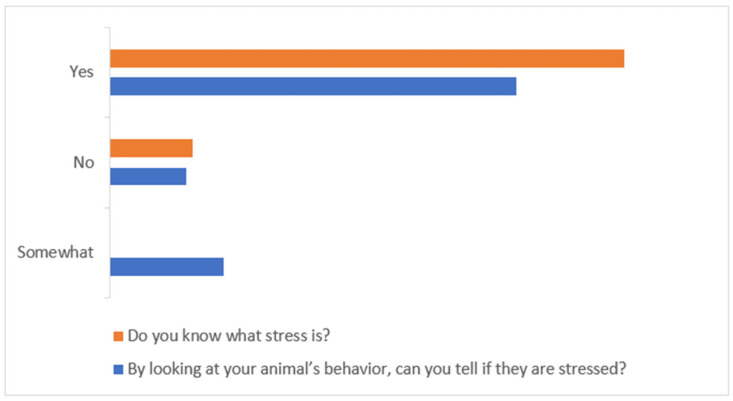
Owners’ opinion about general stress knowledge and the ability to recognize it in their companion animals.

When owners were asked whether they knew what stress is, most of them (86.17%) answered positively. Additionally, more than half (68.09%) believed that they were able to identify specific stress behaviors in their pets, whilst eighteen (19.15%) were unsure, responding with ‘somewhat’ (Figure 2).

### 3.5. Behavioral Analysis During Various Stages of the Veterinary Visit

Table 3 shows the behaviors observed by the owners in their animals at the veterinary practice: at their arrival, right after entering, and after about 10 min spent in the waiting room. For the majority of behaviors identified, the differences between these stages were statistically significant.

Then, the owners were asked about how much stress they noticed in their animals in the same three moments at the veterinary visit. The findings revealed a statistically significant increase in the animals’ stress level over a few minutes: from before entering the veterinary practice to right after entering (*p* < 0.05). Ten minutes later, the results showed a statistically significant reduction in stress (*p* < 0.001).

**Figure 3:**
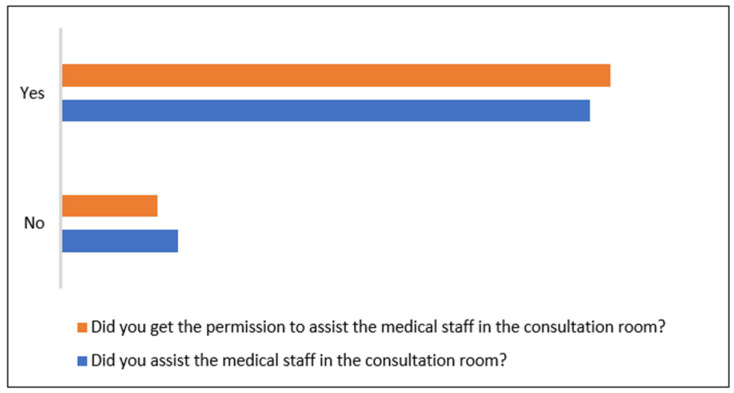
Owners’ attendance at their animals’ consultation.

Most of the owners (85.11%) were allowed by the veterinary practician to assist their animal during consultation, and only 14.89% were not permitted in the consultation room (Figure 3). When the owners were questioned whether they followed the veterinarian in the consultation room, 81.91% responded with ‘yes’, and seventeen owners (18.09%) responded with ‘no’. Despite having the possibility to assist their pets, 3.20% of owners did not want to enter the examination room.

#### 3.5.1. Weighing

**Figure 4:**
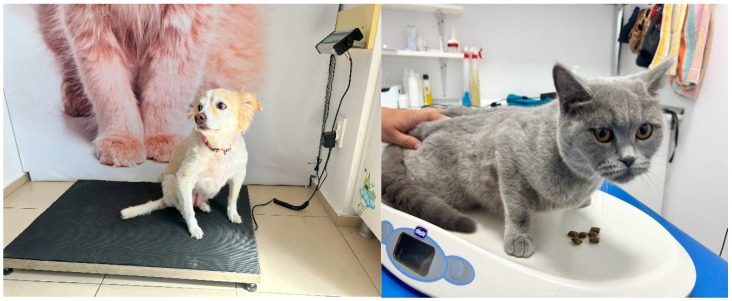
Behavioral patterns: calm or curious (**left**) and alert or attempting escape (**right**).

Regarding their animals’ weighing, one-third of dog and cat owners (30.00%) declared that their animals had calm and cooperative behavior, 21.00% reported escape attempts, and 14.00% observed fear (Figure 4). Less observed behavioral displays included anxiety (7.00%), curiosity (3.00%), agitation/stress (3.00%), trembling (2.00%), energetic/liveliness/happiness (2.00%), and aggressiveness (2.00%). On a scale from 1 (not stressed at all) to 5 (extremely stressed), more than a third of the owners (32.98%) considered that their dog(s) or cat(s) showed a very low level of stress during weighing, followed by low (20.21%), mild (11.70%), moderate (8.51%), and extreme (3.19%) levels of stress. A total of 23.41% of companions were not seen by their owners as they were being weighed.

#### 3.5.2. Entering the Consultation Room

**Figure 5:**
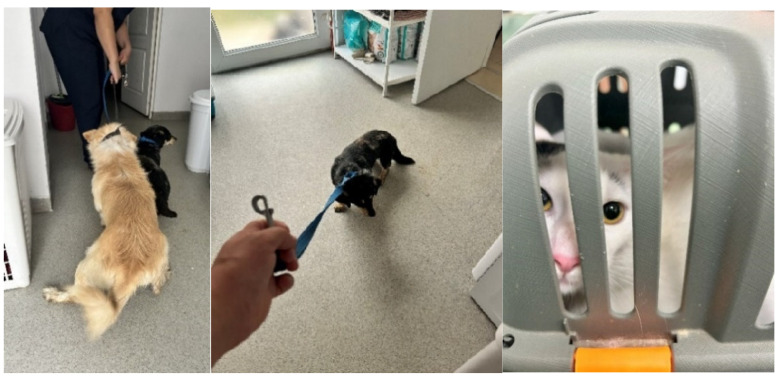
Patient severely stressed by the handler (**left**) or refusing to move (**middle**), and dilated pupils suggesting a high state of alert (**right**).

The survey results indicated that, on a scale from 1 (not stressed at all) to 5 (extremely stressed), a considerable proportion of owners (28.72%) considered that their animal experienced very low stress levels, closely followed by low (20.21%), mild (25.53%), moderate (18.09%), and extreme (6.38%) stress levels (Figure 5). Only one person (1.07%) was not present when the animal entered the consultation room.

#### 3.5.3. Handling

Both dogs and cats were handled either by a veterinarian or a student during consultations. According to the owners’ answers, the majority of animals (88.30%) did not display any signs of aggressiveness at first approach or during handling, whilst five did (5.32%) and six owners replied ‘somewhat’ (6.38%), being uncertain.

On a scale from 1 (impossible to manipulate) to 5 (extremely manipulable), a significant proportion of dogs and cats were considered by their owners to be very manipulable (24.47%) and manipulable (35.11%) during the veterinary visit. Eleven (11.70%) animals could be handled moderately, six (6.38%) were difficult to manipulate, and another six (6.38%) were impossible to manipulate during consultations. A proportion of 15.96% of the owners did not know how to answer or were not present at the consultation. Some examples of handling can be seen in Figure 5.

#### 3.5.4. Home Environment

A considerably broad list of behaviors was provided to the owners, and they were asked to select all descriptors seen in their animal(s) during their daily routine at home. Therefore, one or more behaviors were identified in each patient who visited the veterinary practice.

**Figure 6:**
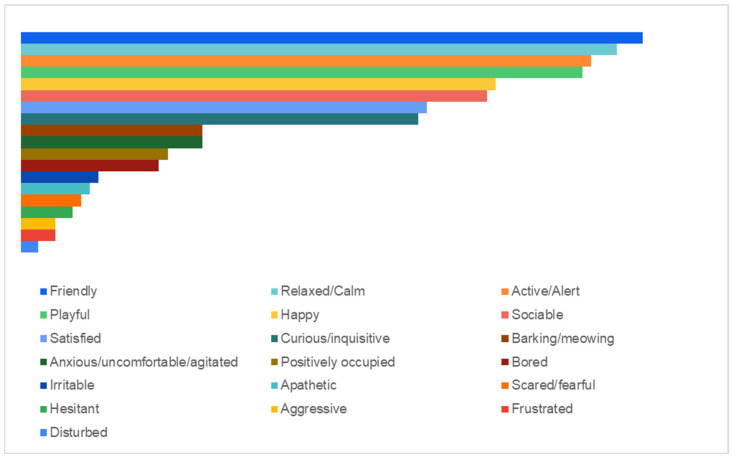
Distribution of most commonly encountered behaviors in dogs’ and cats’ daily home routines.

The findings showed that a significant proportion of dogs and cats (76.60%) displayed friendly behavior, closely followed by relaxed/calm (73.40%), active/alert (70.21%), playful (69.15%), happy (58.51%), sociable (57.45%), thankful/satisfied (50.00%), and curious/inquisitive (48.94%) behaviors. Barking and meowing (vocalizations) were reported by almost a quarter of owners (22.34%) in their animals’ daily routine, followed by positively occupied (18.09%), bored (17.02%), anxious/uncomfortable/agitated (12.77%), nervous (9.57%), irritable (9.57%), apathetic (8.51%), scared/fearful (7.45%), hesitant (6.38%), aggressive (4.26%), and disturbed (2.13%) behaviors (Figure 6).

#### 3.5.5. Behavior During the Veterinary Visit

**Figure 7:**
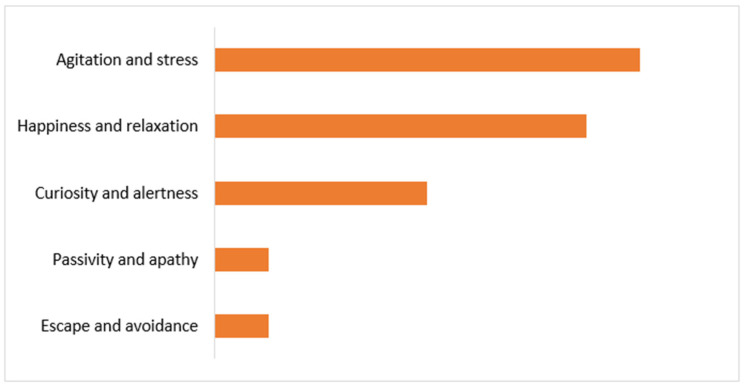
Distribution of the most observed behaviors during the veterinary consultation (top 5).

After identifying the dominant daily behavioral patterns in the studied dogs and cats, the main focus shifted to labeling the most visible behaviors observed by the owners from the moment they left their home until the end of the veterinary visit (Figure 7).

**Figure 8:**
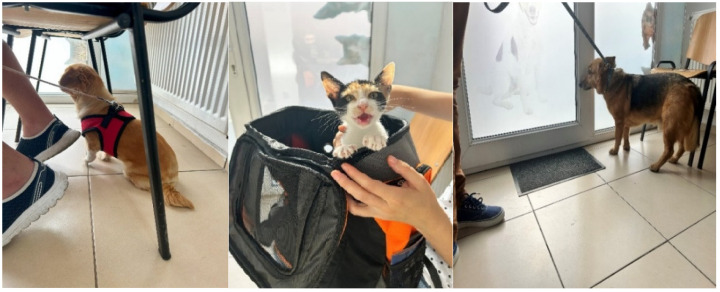
Hiding under owner’s chair as an obvious sign of stress (**left**), presence of vocalizations (**middle**), and escaping attempt (**right**).

As the owners declared, the five most commonly seen behaviors since they had left their homes and until the end of the veterinary consultation were agitation and stress (38.10%, Figure 8), happiness and relaxation (33.30%), curiosity and attention (19.00%), escape attempts and avoidance (4.80%), and passivity and apathy (4.80%), as shown in Figure 7.

### 3.6. Pain Level Assessment

Animals’ pain levels as described by their owners varied considerably. More than a third of them (37.23%) considered their dogs or cats as having a very reduced level of pain (no pain at all), a total of 17.02% as having reduced pain, 11.70% as having mild pain, 6.38% as having moderate pain, and two of them (2.12%) as having a very high level of pain (extreme pain). More than a quarter (25.55%) of the owners did not know the answer or did not participate in the consultation.

### 3.7. Owners’ Perception of Their Pets’ Welfare and the Medical Team’s Approach

**Figure 9:**
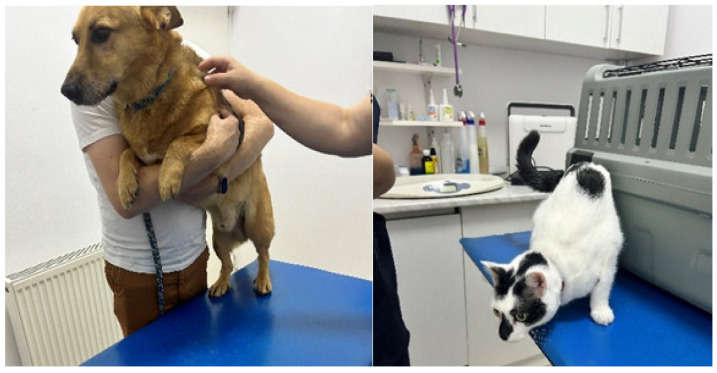
Behavioral signs of fear/insecurity (**left**) and escape attempt (**right**) during veterinary consultation.

Owners were surveyed about the welfare benefits of accompanying their animals in the consultation room. On a scale from 1 (not beneficial at all) to 5 (extremely beneficial), a considerable proportion of respondents (76.6%) considered it beneficial to assist their companion during the veterinary consultation. Apart from that, eleven (11.7%) gave a score of 4, eight (8.5%) gave a score of 3, one (1.1%) gave a score of 2, and two (2.1%) gave the lowest score possible. Some examples of owners assisting their pets during the veterinary consultation can be seen in Figure 9.

According to the survey, a total of 87.23% considered that the welfare of their animal was not compromised during the routine veterinary consultation, whilst a proportion of 11.70% of the owners did not participate in the consultation and one of them (1.07%) did not know how to answer.

Despite that, when owners were asked about what they think are the main issues impacting animal welfare during a routine veterinary consultation, 20.20% of them named stress and fear, followed by the quality and attitude of medical staff (19.10%), presence of other animals and people (11.70%), other miscellaneous situations (9.60%), and the environment or infrastructure, such as space allowance or the lack of specifically designated areas for only dogs or cats (9.60%). Although 21 owners (22.30%) could not recognize any problems or did not know what to answer, interestingly, seven of them (7.50%) indicated that the presence of the owner could harm the welfare of their dogs and cats.

**Figure 10:**
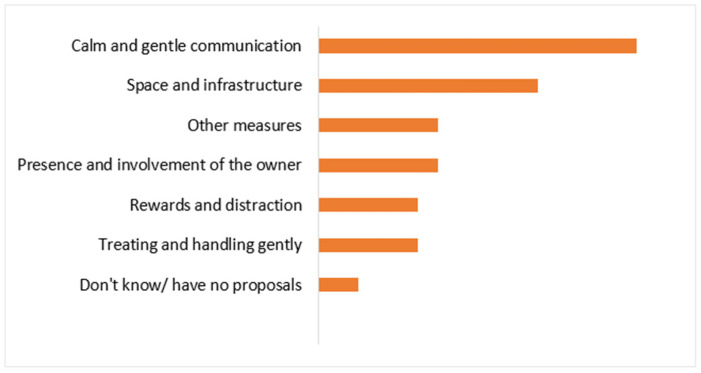
Percentage share of measures suggested by respondents to improve animal welfare during a routine veterinary consultation.

To explore the crucial role of pet owners in ensuring the welfare of companion animals during veterinary visits, they were asked to suggest preventative or ameliorating measures to improve animal welfare during routine veterinary consultations. Two key factors guided this approach: firstly, owners are often the best judges of their pets’ needs and emotions; secondly, animals tend to behave differently in the presence of their guardians or in familiar environments. According to 31.37% of the respondents, calm and gentle communication was the most important element in the veterinarian–patient relationship. Additionally, 21.57% of owners highlighted the importance of sufficient space and adequate infrastructure. Other suggested measures included unspecified actions (11.76%), owner presence and involvement (11.76%), rewards or distractions (9.80%), and gentle handling or treatment (9.80%). A small percentage (3.94%) of respondents were unsure or had no specific recommendations (Figure 10).

Regarding the approach of the medical staff towards clients, most owners (91.49%) declared that the veterinarian was committed to acting for the welfare of their dog or cat, and a total of 72.34% were sociable and open, providing clear information. None of the respondents had a negative experience in terms of veterinary services during their visit. This result was also supported by 93.62% of the answers declaring that the welfare of their companion animal was not compromised during the consultation. Four owners (4.26%) felt the opposite, while two (2.12%) were undecided.

**Figure 11:**
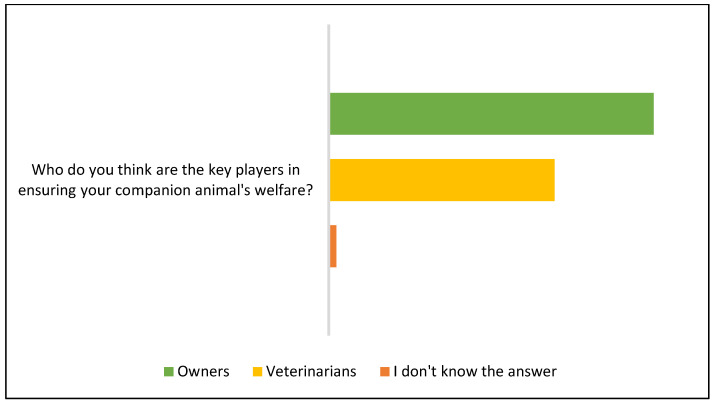
Owners’ perception regarding the shared contribution for their animals’ welfare during routine veterinary visits.

Additionally, more owners attributed a bigger role to themselves than to veterinarians in ensuring their pets’ welfare (90.43% and 62.77%, respectively), whilst two (2.13%) did not know the answer (Figure 11).

## 4. Discussion

This study explored Romanian pet owners’ perceptions of their animals’ behavior and welfare during veterinary visits and provided complementary insights to existing research on animal welfare. The survey indicated that most veterinary clients owned dogs. Previous studies show that more than half of veterinary appointments for dogs and cats involve routine vaccinations and parasite control, while over a third are for other medical issues [11]. Similarly, our results revealed that owners primarily scheduled appointments for routine health check-ups due to their interest in keeping their pets healthy and recognizing the importance of timely diagnostics.

Regarding pet demographics, the survey found no significant gender differences in the studied pet species. Similar figures were shown by Vizcaíno et al. [14], describing an almost equal sample of female dogs and cats visiting veterinary practices. However, the age distribution showed that the smallest groups visiting veterinary practices were animals older than eight years, followed closely by those under one year of age. In contrast, a large-scale British study found that young dogs and cats (under one year) were the least represented group in veterinary visits [14]. While nearly a decade has passed since that study, our findings suggest a gradual increase in the owners’ awareness to seek veterinary attention for young pets and a decline in older-pet visits. Despite cultural and geographical differences, reports such as the FVE’s 3rd VetSurvey highlight a general shift in veterinary care from farm animals to companion animals. While we did not compare the market demand in this regard, the increased presence of young dogs and cats at veterinary practices may also signal an increase in the number of pet owners.

According to the survey responses, owners showed a preference for keeping either one or multiple pets of various species and emphasized the importance of access to open spaces such as parks or gardens. A study on pet ownership in the UK similarly found that most owners had access to a garden [15]. Housing conditions play a crucial role in managing stress during veterinary visits, underscoring the need to consider factors beyond the clinic setting when evaluating animal welfare.

Owner–pet interaction also appeared to be a key factor. Romanian pet owners reported spending an average of 14 to 35 h per week with their pets, compared to American owners, who spend approximately 45.3 h with dogs and 32 h with cats weekly [16]. Owners also noted frequent interactions between their pets, and some chose to bring all their animals to the veterinarian together, believing that the presence of companions can mutually reduce their stress. On the other hand, indeed, strong bonds between animals can lead to separation anxiety when one is left behind.

Most owners considered that they knew what stress is and assessed themselves to be able to recognize stress-related behaviors in their animals. A study by Beaver [17] found that many dogs exhibit submissive behaviors linked to fear during veterinary visits. In contrast, cats are typically transported in carriers, with those unfamiliar with travel vocalizing more, particularly males [18]. Research by Mariti et al. [19] report that over two-thirds of cats are brought to the veterinarian in carriers, while a small percentage are held or leashed. Certain carrier designs, particularly those without a detachable top, can make handling difficult, prolonging consultations and increasing the animals’ stress. Recommendations from international organizations suggest using solid plastic carriers with detachable lids and applying enrichment techniques, such as Feliway spray, to improve comfort and reduce stress.

For canine patients, Lind et al. [20] report that more than half of those entering veterinary practices exhibit fear or anxiety, according to their owners. In our study, unfamiliar environments and stimuli (e.g., the presence of other animals) contributed to initial stress spikes, from arrival at the veterinary practice to the moment right after entering. While behaviors such as trembling and vocalization decreased over time, after about 10 min spent in the waiting room, signs of curiosity, relaxation, and happiness suggested that some animals adapted to the setting. However, stress-related behaviors persisted in certain individuals even during and after the consultation. To minimize stress, some veterinarians recommended owners to wait outside instead of the waiting room, to reduce their pet’s exposure to other animals. Another beneficial option would be for small animal veterinary practices to have separate entrances and waiting areas (or rooms) for dogs and cats.

Owner assistance during the consultation varied in this study; some were not allowed to assist, while others were permitted to stay. In some cases, a lack of clear communication led to misunderstandings about the veterinary team’s willingness to involve owners. Excluding owners without justification should be avoided through better communication at the start of consultations. Communication problems with clients can seriously impact the credibility of veterinary professionals, leaving owners susceptible to misunderstanding them.

Regarding stress levels, many clients rated the stress of their dogs and cats as low to mild during weighing. In contrast, Hammerle et al. [21] found that about half of dogs showed increased stress when weighed. Hernander [22] suggested that weighing dogs before owners enter the consultation room can reduce stress. For cats, weighing in the carrier, partially covered by a blanket, can help minimize stress [23]. Our study supports these findings, indicating that stress levels depend on various circumstances, in addition to the individual traits of each animal. In an interesting study, Döring et al. [24] found that nearly half of dogs entered the consultation room easily, on their leash, without being forced. Mariti et al. [19] noted that cats often appeared disturbed when moving from the waiting area to the consultation room. In our study, the owners did not observe signs of aggression in their animals during handling. Glardon et al. [25] found similar levels of aggression in dogs and cats during examination. A recent study suggested that dogs were less stressed during exploration of the consultation room and that the presence of owners had a positive effect on their behavior [26].

While most dogs and cats did not show aggression, our findings align with research suggesting that around 22% of dogs and cats could be handled only partially during consultations, with stress and elevated cortisol levels as contributing factors [27]. Cats, being more difficult to handle due to their nature, require calming strategies to minimize fear. Rodan et al. [28] emphasized that cat-friendly visits should provide a sense of control to reduce negative experiences. Additionally, grouping animals by species during appointments can reduce stress, anxiety, and vocalizations. Studies also showed that minimal restraint methods reduce fear compared to more invasive techniques [29]. A proper stress-management strategy is crucial during veterinary visits, even for routine consultations, as stress is closely linked to pain. Some owners felt their pets experienced varying levels of pain. A Canadian study found that 65% of pet owners disagreed that pain in animals is easily recognizable [30].

Kogan et al. [31] found that nearly 97% of owners felt respected and cared for by veterinary staff, which our study supports. More than 90% of the studied owners declared that the veterinarian showed commitment to protect the welfare of their animals during consultation, and no responder reported negative experiences. This shows a considerably high level of owner respect and trust in their relationship with veterinary personnel.

The implications and impact of this research are wide and involve veterinarians, veterinary practices, pet owners, and their pets. By understanding owners’ perceptions, veterinarians can anticipate certain undesirable behaviors and adjust their protocol according to pets’ needs. Although communication between owners and veterinarians can sometimes be difficult, a good veterinarian–owner relationship is pivotal to ensuring pets’ welfare. Veterinary practices could implement various enrichment techniques to make owners’ and pets’ visits to the practice a positive experience. Some examples, such as designated species-specific waiting rooms, pre-visit medication (whenever required), and the use of low-stress handling techniques, have been found successful even in the most fearful patients. Owners’ education must be focused on the latest recommendations that will allow them to recognize normal behavior from abnormal behavioral patterns. The implementation of measures aiming to minimize stress-related behaviors, therefore, could lead to better welfare outcomes by improving the quality of veterinary consultations.

## 5. Conclusions

Assessing the behavior of dogs and cats can be challenging, especially when resources are limited. Using behavioral indicators to assess animal welfare can provide valuable insights into their condition and help develop behavioral profiles for patients, potentially improving undesirable behaviors that affect welfare. According to owners, stress, fear, staff interaction, and the presence of others during consultations were key welfare concerns. Important factors for ensuring proper welfare include calm communication, proper infrastructure, humane handling, and offering treats. Poor-quality visits can significantly impact the behavior and welfare of companion animals. Veterinarians must educate owners on how to reduce stress in their pets before visits. Providing high-quality consultations can have a positive effect on animals’ physical and mental well-being by reducing stress and anxiety, leading to more efficient consultations and a safer environment for animals and owners. While this study was well received by owners, further research on companion animal behavior and welfare is crucial for developing strategies to mitigate stress during veterinary visits. Future studies could potentially focus on the investigation of the impact that various handling techniques have on owners’ perception in connection with pets’ behavior, while organizing different events such as workshops and training schools, providing useful online resources, and offering valuable veterinary consultations could contribute to the education of owners in recognizing and managing stress in their pets effectively before veterinary visits. This is the reason why social media campaigns and reliable informational materials are essential for animals to benefit from a positive experience whenever visiting a veterinarian. Therefore, owners should be aware that stress behaviors are a common issue in dogs and cats during a consultation, but they can be substantially minimized with reduced input. On the other hand, veterinary medical teams should provide information about the means by which all these results can be achieved effectively (e.g., carrier training, desensitization) and consider the implementation of fear-free veterinary (handling) techniques that support pet welfare. By implementing and incorporating these measures, routine veterinary visits would benefit from high welfare standards and improved owner satisfaction.

## Figures and Tables

**Table 1:** General information of the studied dogs and cats.

Demographic Data			
		*n*	%
Dog	All dogs	55	58.51
	Male dogs	30	54.54
	Female dogs	25	45.46
Cat	All cats	39	41.49
	Male cats	19	48.72
	Female cats	20	51.28
Age	Younger than 1 year old	20	23.24
	1–8 years old	54	56.67
	Older than 8 years old	16	17.78
	Uncategorizable	4	2.31
Age by species	1–8-year-old dogs	35	63.64
	1–8-year-old cats	19	54.28
	Older than 8 years old (dogs)	12	21.81
	Older than 8 years old (cats)	4	11.43

**Table 2:** The frequency of management-related and social characteristics of the studied dogs and cats.

Demographic Data			
Type of Housing		*n*	%
	Apartment	49	53.85
	House	38	41.76
	Both options	4	4.39
Number of animals in a household	Different species	12	26.67
	At least one dog	7	15.56
	At least one cat	10	22.23
	Two dogs	5	11.12
	Two cats	4	8.89
	More than two dogs	3	6.67
	More than two cats	3	6.67
Space allowance	Garden or park	72	79.12
	Not provided	22	20.88
Human–animal interaction	Frequent interaction	89	97.80
	Infrequent interaction	2	2.20
Outdoor access allowance	Full-day access	29	37.18
	Less than 2 h per day	20	25.64
	2–5 h per day	14	17.95
	More than 5 h per day	9	11.54
	No outdoor access	6	7.69
Animal–animal interaction	Frequent	37	41.57
	Infrequent	8	8.99
	Absent (no other animal in the household)	44	49.44
Perceived abnormalities (overall)	None perceived	74	81.32
	Perceived	15	16.48
	Unsure	2	2.20

**Table 3:** Most prevalent behaviors observed by the 94 dog and cat owners over different phases of veterinary consultation.

Behavior	Before Entering the Veterinary Practice (%)	Right After Entering the Veterinary Practice (%)	Ten Minutes After Entering the Veterinary Practice (%)	*p*-Value (Friedman Test)
Fearful	0	19.84	3.64	<0.001
Trembling	19.51	14.26	6.36	<0.001
Attempting escape	13.82	11.12	3.64	<0.001
Vocalizing	11.38	7.94	5.45	<0.001
Displaying other behaviors	6.50	0	0	<0.001
Hyperexcitable	3.25	2.38	0	0.039
Agitation	3.25	7.94	8.18	0.006
Refusal to enter the practice	3.25	0	0	0.018
Insecurity	3.25	3.97	0	0.015
Tail tucking between legs	2.44	0	0	0.05
Calm or relaxed	2.44	14.00	13.64	<0.001
Curious	0	10.32	10.00	<0.001
Shy	0	3.17	0	0.018
Anxious	0	3.17	2.73	0.039
Apathetic	0	1.59	0	0.135
Joyful or happy	0	1.59	0	0.135
Aggressive	0	0.79	0	0.368
Actively playing	0	0	3.64	0.018

If a *p*-value is less than 0.05, the difference between assessments is significant.

## Data Availability

Data sharing is applicable to this article, and the data are available upon request from the authors.

## Supplementary Materials

The following supporting information can be downloaded at: https://www.mdpi.com/article/10.3390/ani15060894/s1, File S1: Questionnaire.
