# Supplementary material for: Pet Owners’ Perceptions of Key Factors Affecting Animal Welfare During Veterinary Visits
Source: Animals (Basel). 2025 Mar 20;15(6):894. doi: 10.3390/ani15060894 (PMC11939186; doi:10.3390/ani15060894)
Supplement: Supplementary file 1 [file animals-15-00894-s001.zip › animals-3509513-supplementary.pdf]

animals

MDPI

Pet owners’ perceptions of key factors affecting animal welfare during veterinary visits

Owner questionnaire

Q1: I, the undersigned, owner/handler of the animal mentioned below, declare that I agree to participate in this study.

A1: Yes

Q2: Date of filling out the questionnaire:

A2: …

Q3: What is the reason for your appointment at this veterinary unit? E.g. routine consultation, vaccination and/or deworming, symptoms of disease (please describe), other (please describe).

A3: …

Q4: The species of your pet is:

A4: Dog / Cat

Q5: What is the name of your pet?

A5: …

Q6: What is the age of your pet? (in month for younger than one year old, in years for older)

A6: …

Q7: What is the sex of your pet?

A7: Male / Female

Q8: Is your pet neutered?

A8: Yes / No

Q9: Do you know what stress is?

A9: Yes / No

Q10: By looking at your animal’s behavior, can you tell if they are stressed?

A10: Yes / No / Sometimes

Q11: Does your pet have daily free outdoor access at home?

A11: Yes / No

Q12: If yes, how much time per day (hours, minutes)?

A12: …

Q13: If no, please explain why.

A13: …

Q14: Do you have any other animal(s) in the household?

A14: Yes / No

Q15: If you have other dog(s) or cat(s), please write how many of them.

A15: … dogs, … cats

Q16: Do you have daily one-to-one interaction with this pet?

A16: Yes / No

Q17: If yes, please write how much time per day (hours, minutes).

A17: …

Q18: If no, please write how often you interact with this pet and for how long (hours, minutes).

A18: …

Q19: If you have other dog(s) and/or cat(s), do they interact freely at home?

A19: Yes / No / I do not have other dog(s) / cat(s)

Q20: Did you notice any abnormal behavior in your pet?

A20: Yes / No / I do not know

Q21: If yes, please describe briefly what you noticed.

A21: …

Q22: What behaviors did your pet show before entering the veterinary unit (e.g., fearful, trembling, attempting escape, vocalizing, hyperexcitable, agitation, refusal to enter the practice, insecurity, tail tucking between legs, calm or relaxed, curious, shy, anxious, apathetic, joyful or happy, aggressive, actively playing or other behaviors)? Please describe briefly.

A22: …

Q23: What behaviors did your pet show right after entering the veterinary unit (e.g., fearful, trembling, attempting escape, vocalizing, hyperexcitable, agitation, refusal to enter the practice, insecurity, tail tucking between legs, calm or relaxed, curious, shy, anxious, apathetic, joyful or happy, aggressive, actively playing or other behaviors)? Please describe briefly.

A23: …

Q24: Compared to the behaviors you observed in your pet right after entering the veterinary unit, did your pet display these more or less after 5-10 minutes in the waiting room (e.g., more or less fearful, trembling more or less, attempting escape more or less, vocalizing more or less, etc.)? Please describe briefly.

A24: …

A25: When you arrived at the veterinary unit, what stress level did your animal display? Please rate on a scale from 0 to 5, where 0 is "Not stressed at all" and 5 is "Extremely stressed".

A25: …

Q26: Right after entering the veterinary unit, what stress level did your animal display? Please rate on a scale from 0 to 5, where 0 is "Not stressed at all" and 5 is "Extremely stressed".

A26: …

Q27: After about 10 minutes in the waiting room, what stress level did your animal display? Please rate on a scale from 0 to 5, where 0 is "Not stressed at all" and 5 is "Extremely stressed".

A27: …

Q28: Were you allowed to accompany your animal in the consultation room?

A28: Yes / No

Q29: Did you accompany your animal in the consultation room?

A29: Yes / No

Q30: What behavior did your animal show when it was weighed?

A30: … / I was not present at its weighing

Q31: What stress level did your animal display at its weighing? Please rate on a scale from 1 to 5, where 1 is “Not stressed at all” and 5 is “Extremely stressed”. Please choose 0 if you were not present at its weighing.

A31: …

Q32: What stress level did your animal display when entering the consultation room? Please rate on a scale from 1 to 5, where 1 is “Not stressed at all” and 5 is “Extremely stressed”. Please choose 0 if you were not present when your animal entered the consultation room.

A32: …

Q33: Did your pet show signs of aggression when approached/handled by the veterinarian/student?

A33: Yes / No / Somewhat

Q34: How manageable was your pet during the consultation? Please rate on a scale from 1 to 5, where 1 is “Impossible to manipulate” and 5 is “Extremely manipulable”. Please choose 0 if you were not present with your animal in the consultation room.

A34: …

Q35: What was the stress level of your animal during the veterinary consultation? Please rate on a scale from 1 to 5, where 1 is “Not stressed at all” and 5 is “Extremely stressed”. Please choose 0 if you were not present with your animal in the consultation room.

A35: …

Q36: Which of the following behaviors did you observe in your animal at home, in your daily routine? Please choose all that apply.

A36: Friendly; Playful; Satisfied; Anxious/Uncomfortable/Agitated; Irritable; Hesitant; Disturbed; Relaxed/Calm; Happy; Curious/Inquisitive; Positively occupied; Apathetic; Aggressive; Active/Alert; Sociable; Barking/Meowing; Bored; Scared/Fearful; Frustrated

Q37: What were the Top 5 most pronounced behaviors you observed since leaving your home until the end of the consultation? Please assign a level of intensity from 1 (the lowest) to 10 (the highest).

A37: …

Q38: How much pain do you believe your pet felt during the consultation? Please rate on a scale from 1 to 5, where 1 is “No pain at all” and 5 is “Extreme pain”. Please choose 0 if you were not present with your animal in the consultation room.

A38: …

Q39: How beneficial was your presence in the consultation room for your animal's welfare? Please rate on a scale from 1 to 5, where 1 is “Not beneficial at all” and 5 is “Extremely beneficial ”. Please choose 0 if you were not present with your animal in the consultation room.

A39: …

Q40: Please briefly describe the main issues impacting the welfare of your animal during the veterinary visit.

A40: ...

Q41: Please describe briefly a preventative or ameliorating measure to safeguard animal welfare during veterinary visits.

A41: ...

Q42: How would you rate the approach of the medical staff at the practice?

A42: Involved and willing to help; Sociable, open, and honest with the information provided; Careless/disinterested/non-communicative; Hesitant to provide details about my pet's condition

Q43: Was your pet's welfare compromised during the consultation?

A43: Yes / No / Somewhat

Q44: Who do you think are the main players in ensuring your pet's welfare during the veterinary consultation?

A44: Owners / Veterinarians / I do not know the answer

Q45: Please rate this questionnaire on a scale from 1 (not interesting at all) to 5 (very interesting).

A45: …

Q46: Please add any other comments you would like to share.

A46: …

Q47: I agree and acknowledge that the personal data contained in this form are processed in accordance with the applicable laws and treated confidentially, in compliance with Regulation (EU) 2016/679 on the protection of individuals concerning the processing of personal data and on the free movement of such data, repealing Directive 95/46/EC (General Data Protection Regulation).

A47: Yes / No
